# Sex-related differences in the efficacy and toxicity of cancer treatments

**DOI:** 10.1007/s12094-025-03893-2

**Published:** 2025-03-28

**Authors:** Ana Santaballa Bertrán, José Antonio Marcos Rodríguez, Ana Cardeña-Gutiérrez, Virginia Martinez-Callejo, Oliver Higuera, Beatriz Bernardez, Maria-Estela Moreno-Martínez, Margarita Majem

**Affiliations:** 1 Department of Medical Oncology, La Fe University Hospital, IISLaFe, Valencia, Spain; 2 Oncology Pharmacy Unit, Pharmacy Service, Virgen Macarena University Hospital, Sevilla, Spain; 3 Department of Medical Oncology, Nuestra Señora de Candelaria University Hospital, Carretera General del Rosario, 145, 38010 Santa Cruz de Tenerife, Spain; 4 Oncology Pharmacy Unit, Pharmacy Service, Marqués de Valdecilla University Hospital, Avda Marqués de Valdecilla, S/N 39008, Santander, Spain; 5 Department of Medical Oncology, La Paz University Hospital, Madrid, Spain; 6 Departament of Medicine and Pharmacology Group, University of Santiago de Compostela, Santiago de Compostela, Spain; 7 Oncology Pharmacy Unit, Pharmacy Service, University Clinic Hospital of Santiago de Compostela, Santiago de Compostela, Spain; 8 Santiago de Compostela Research Institute (IDIS), Santiago de Compostela, Spain; 9 Pharmacy Department, Santa Creu I Sant Pau Hospital, IIB Sant Pau, Barcelona, Spain; 10 Blanquerna School of Health Sciences, University Ramon Llull, Barcelona, Spain; 11 Department of Medical Oncology, Santa Creu I Sant Pau Hospital, IIB Sant Pau, Barcelona, Spain

**Keywords:** Cancer, Antineoplastic agents, Radiotherapy, Sex, Survival, Toxicity

## Abstract

Differences between the biological sexes have long been observed in cancer incidence and prevalence, and in treatment outcomes including efficacy and toxicity. Ideally, there should be sufficient information to improve the individualization of cancer treatment by incorporating sex into treatment decisions. Necessary information should include: the nature and source of these differences; whether inherent to the specific cancer (such as molecular profiles, metabolic behaviors, and immune responses); the pathophysiological mechanisms of the specific cancer; or the pharmacokinetic and pharmacodynamic profiles of different cancer drugs. The influence of gender, which is defined as the sociocultural construct that determines societal norms for males and females, should also be included in personalized decision-making. This review aimed to describe the current evidence on the impact of sex and gender on treatment effects, outcomes, and toxicity profiles in cancer patients. Data for the influence of gender were negligible, whereas clinical studies and meta-analyses in different cancer types have identified differences between males and females in the effectiveness on survival outcomes of immunotherapy, chemotherapy, targeted therapy, and radiotherapy. Similarly, toxicity profiles of different cancer treatments varied between sexes. Based on these observed differences, it seems clear that sex should be included as an important variable when individualizing treatment; however, more research into sex- and gender-related differences in cancer treatment efficacy and toxicity, and the causes for these differences, is required before this can be fully incorporated into individualized treatment programs in real-world clinical practice.

## Introduction

There is now considerable evidence that the pathophysiological mechanisms of cancer differ between males and females [1], and that these differences account for disparities in the incidence and prevalence of non-reproductive cancers between the sexes [2–4]. In addition, gender (the sociocultural construct that determines societal norms for males and females) may influence health behaviors and healthcare access, affecting cancer epidemiology and outcomes [5].

There are differences between the sexes in the molecular profiles, metabolic behaviors, and immune responses of specific cancers (reviewed by Bernardez et al. [6]), which may translate into differences in treatment effects [1]. Moreover, the pharmacokinetic and pharmacodynamic behaviors of drugs differ between males and females as a result of different body composition and receptor expression [7], which may affect both efficacy and toxicity. These differences underlie the rationale for considering sex as a separate biological variable in cancer, and using sex to individualize treatment and dosage decisions [5].

Several difficulties arise when assessing the influence of sex as a separate biological variable in cancer research. These include the presence of confounding variables that often differ between males and females, such as average age at diagnosis [8] and socioeconomic factors (e.g., lifestyle behaviors, access to education and healthcare) [5]. Such factors are also particularly relevant to the lesbian, gay, bisexual, transgender, queer, intersex, and asexual (LGBTQIA +) community, a medically underserved population whose gender identities, exposure to risk factors, engagement with healthcare systems, and use of gender-affirming care may further confound sex-related differences in the epidemiology, treatment, and outcomes of cancer [9, 10]. In addition, females have historically been under-represented in cancer clinical trials and sex as a variable has often been omitted from data analyses [11, 12], resulting in current clinical practices largely informed by data in males, and a limited biological understanding of cancer and its treatment among females.

The aim of this narrative review is to examine the impact of sex and gender on treatment effects, outcomes, and toxicity profiles in cancer patients, to better understand how to incorporate sex into individualized treatment decisions.

## Methods

A search of PubMed was conducted in January 2024, first using terms for (“sex” OR “gender”) AND (“cancer” OR “oncol*”), and then combining the results of this search with a range of specific terms relating to treatment (including chemotherapy, targeted therapy, tyrosine kinase inhibitors, immunotherapies, and radiotherapy), outcomes (particularly survival), and toxicity (adverse events [AEs]). The search results were reviewed to identify articles specifically examining any potential sex-related differences in treatment efficacy and/or toxicity. Search results were supplemented by additional articles identified from the bibliographies of retrieved articles, or from ad hoc searches in relation to specific topics.

## Efficacy

### Immunotherapy

A major advance in cancer therapeutics in recent decades has been the development of treatments that harness the immune system against cancer, including immune checkpoint inhibitors (ICIs) and chimeric antigen receptor (CAR)-T cell therapy. Available evidence suggests that the effectiveness of these immunotherapies may vary according to sex; however, there is a lack of data to inform potential gender-based differences in survival outcomes.

In randomized clinical trials, both males and females benefit from ICI treatment, but the magnitude of the benefit differs between the sexes and depends on the comparator [13–21]. In a 2018 meta-analysis of 20 randomized controlled trials that included over 11,000 patients with different types of advanced cancer, the survival benefit with ICI therapy was less marked in females than in males, and the magnitude of the difference between the sexes was similar to the magnitude of difference between patients with programmed death ligand-1 (PD-L1)–positive versus PD-L1-negative tumors [13]. The hazard ratio (HR) for overall survival (OS) with ICIs versus control was 0.72 (95% confidence interval [CI] 0.65–0.79) in males and 0.86 (95% CI 0.79–0.93) in females (*p* = 0.0019 vs males). However, this early meta-analysis has been criticized for pooling studies from a range of cancer types and including studies in which ICI treatment was administered as monotherapy or in combination with chemotherapy, as well as including a range of comparators in the control group, such as chemotherapy alone, targeted therapy, or another type of ICI [18, 20]. Three subsequent meta-analyses comparing ICI therapy with non-ICI comparators showed no difference in OS between males and females [18, 20, 21].

**Fig. 1:**
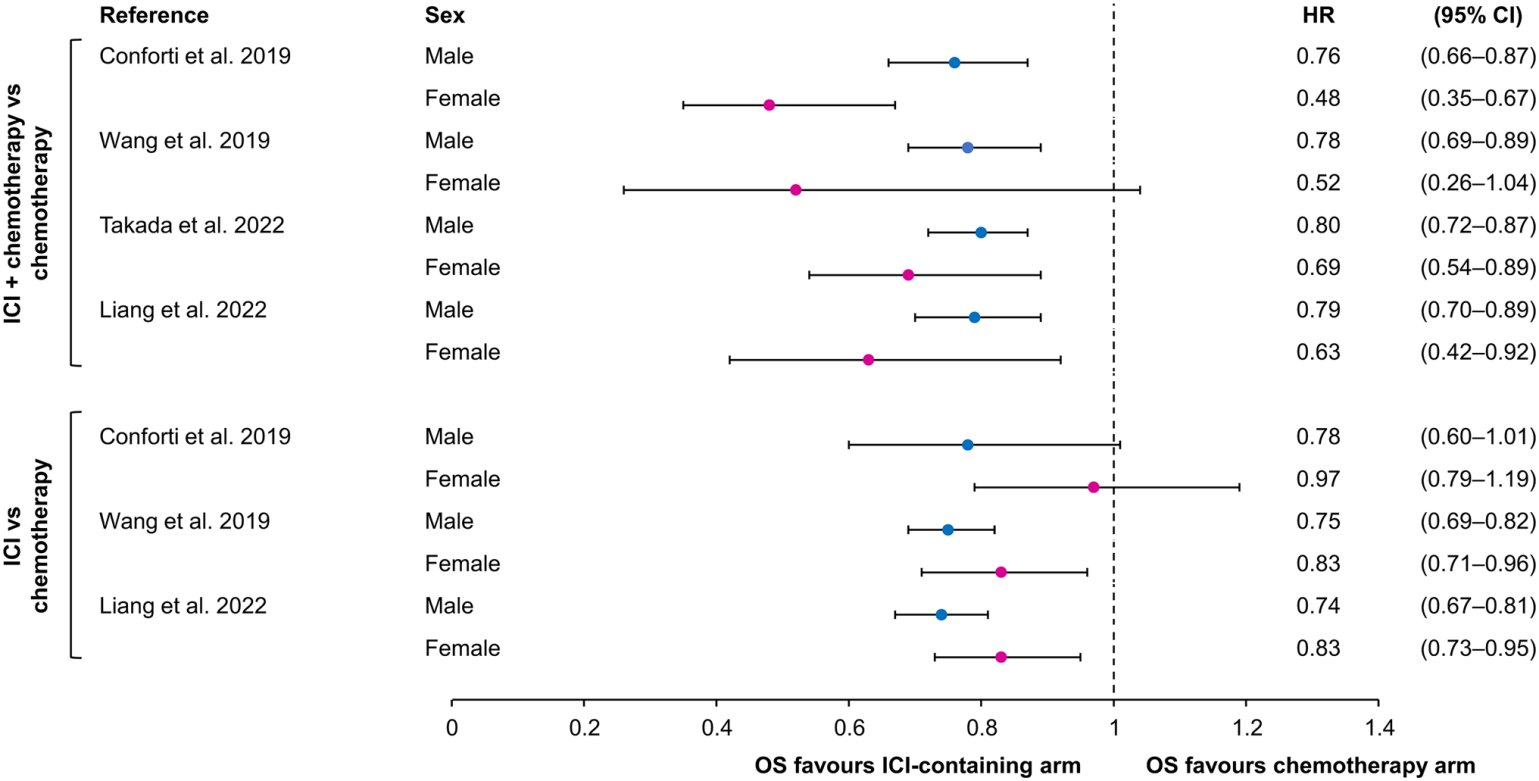
Hazard ratios for overall survival in meta-analyses of immune checkpoint inhibitor therapy among patients with non-small cell lung cancer, stratified by immune checkpoint inhibitor regimen (monotherapy vs combination with chemotherapy) [14, 16, 19, 22]. *CI* confidence interval, *HR* hazard ratio, *ICI* immune checkpoint inhibitor, *OS* overall survival

A key defining factor in the sex difference in ICI efficacy is whether the ICI is administered with chemotherapy or as monotherapy. In meta-analyses of patients with non-small cell lung cancer (NSCLC), there was a greater survival benefit in males than females associated with ICI monotherapy [14–16, 19], whereas females tended to have a more marked survival advantage than males when treated with ICI + chemotherapy combinations (Fig. 1) [14, 16, 19, 22]. This was supported by a real-world analysis of patients with NSCLC, which showed that the survival difference between those receiving ICI + chemotherapy and those receiving chemotherapy without ICI was greater in females than in males, after propensity score matching to adjust for key confounders such as age, comorbidity, and income [17].

The difference in ICI response between males and females is likely to be related to differences in the adaptive and innate immune systems as a result of sex chromosomes and sex hormones [1]. Females mount a more robust acute inflammatory response to infection than males, have higher levels of CD4 + lymphocytes and CD4/CD8 ratios, and show greater T cell activation and proliferation, B cell numbers, and immunoglobulin levels. In contrast, males show higher levels of CD8 + T cells, regulatory T (Treg) cells, and natural killer (NK) cells [23]. The female immune phenotype is associated with greater stimulation of cytotoxic T lymphocytes (CTLs) and a more efficient anti-tumor immune response [23], which may in part explain some sex-related disparities in the incidence and mortality rate of different tumors [24]. Because cancer in females must elude a more ‘vigilant’ immune system, males tend to develop more immunogenic tumors than females [24], while tumors in females tend to be enriched with more efficient mechanisms for immune evasion [23]. The enhanced response to ICIs when combined with chemotherapy in females may be because chemotherapy increases the tumor mutation burden (usually lower in females than males), thus making the cancer more susceptible to ICI treatment when combined with chemotherapy in females [23].

Sex differences in the effects of ICI therapy on survival may also be influenced by other factors such as body composition, tumor histology, and the composition of the gut microbiome [17, 25, 26]. In addition, differential adherence may contribute to differences in outcomes between men and women [27], but to the best of our knowledge, there are no data available comparing ICI adherence between genders.

Relatively less is known about sex-related differences in the response to CAR-T cell therapy, but preliminary results suggest that females with relapsed/refractory large B cell lymphoma may have a better response to this form of treatment than males (data currently available from a congress presentation) [28]. Radiographic response was significantly better in females than in males (*p* = 0.03), with a higher proportion of female patients achieving a complete response and a lower proportion with progressive disease. Progression-free survival (PFS) was significantly longer in females than males (median not reached [95% CI 12.2 to not reached] vs 4.2 months [95% CI 3.2–15.8], respectively; *p* = 0.008) [28]. OS was not different between the sexes.

### Chemotherapy

Sex-related differences in outcomes have been reported for conventional cytotoxic chemotherapy [29], as described here from several studies in various cancers. Most studies reported outcome variables such as OS and PFS.

Data in patients with esophageal cancer or NSCLC show better chemotherapy outcomes in females than males. A pooled analysis of data from four studies in patients with esophageal cancer receiving chemotherapy reported significantly better OS in females than in males (HR 0.83 [95% CI 0.72–0.96]; *p* = 0.011) [30]. PFS also tended to be better in females than males, but the difference did not reach statistical significance (HR 0.87 [95% CI 0.76–1.00]; *p* = 0.06) [30]. A retrospective analysis of trials funded by the National Cancer Institute of Canada found that, after adjustment for confounders, PFS with chemotherapy was significantly better in female than male patients with NSCLC (HR 0.83 [95% CI 0.71–0.97]; *p* = 0.02), but OS was not (HR 0.89 [95% CI 0.75–1.05]; *p* = 0.17) [31].

On the other hand, males may have better outcomes than females in response to the 5-fluoropyrimidines, such as capecitabine and 5-fluorouracil. The BILCAP study in patients with biliary cancer showed that only males achieved a benefit from adjuvant capecitabine after surgery compared with the observation group [32]. HR for OS in males was 0.70 (95% CI 0.50–0.99) compared with 0.93 (95% CI 0.64–1.35) in females [32]. Similarly, males had better outcomes than females in the SOLSTICE study, which compared trifluridine/tipiracil + bevacizumab with capecitabine + bevacizumab in patients with unresectable metastatic colorectal cancer ineligible for full-dose doublet or triplet chemotherapy [33, 34]. Male patients experienced significantly longer median PFS with trifluridine/tipiracil + bevacizumab than with capecitabine + bevacizumab (10.3 months [95% CI 9.2–11.2] vs 9.2 months [95% CI 7.8–9.7], respectively; HR 0.75 [95% CI 0.61–0.93]) whereas female patients experienced no significant PFS advantage (HR 1.02 [95% CI 0.81–1.30]) [34]. In this instance, the sex difference in outcomes was probably influenced by pharmacokinetic and pharmacodynamic factors [35], which are marked for the 5-fluoropyrimidines such as capecitabine and 5-fluorouracil [29]. The 5-fluoropyrimidines show lower elimination in females than males but higher toxicity, while pharmacokinetics are influenced by body composition, indicating that sex-specific dosages should be based on fat-free mass rather than on traditional anthropometric measurements [29].

### Targeted therapies

Targeted therapies also show differences in outcomes between male and female patients. However, most analyses have been conducted in patients with NSCLC, so whether these findings are translatable to other cancers is unclear.

**Fig. 2:**
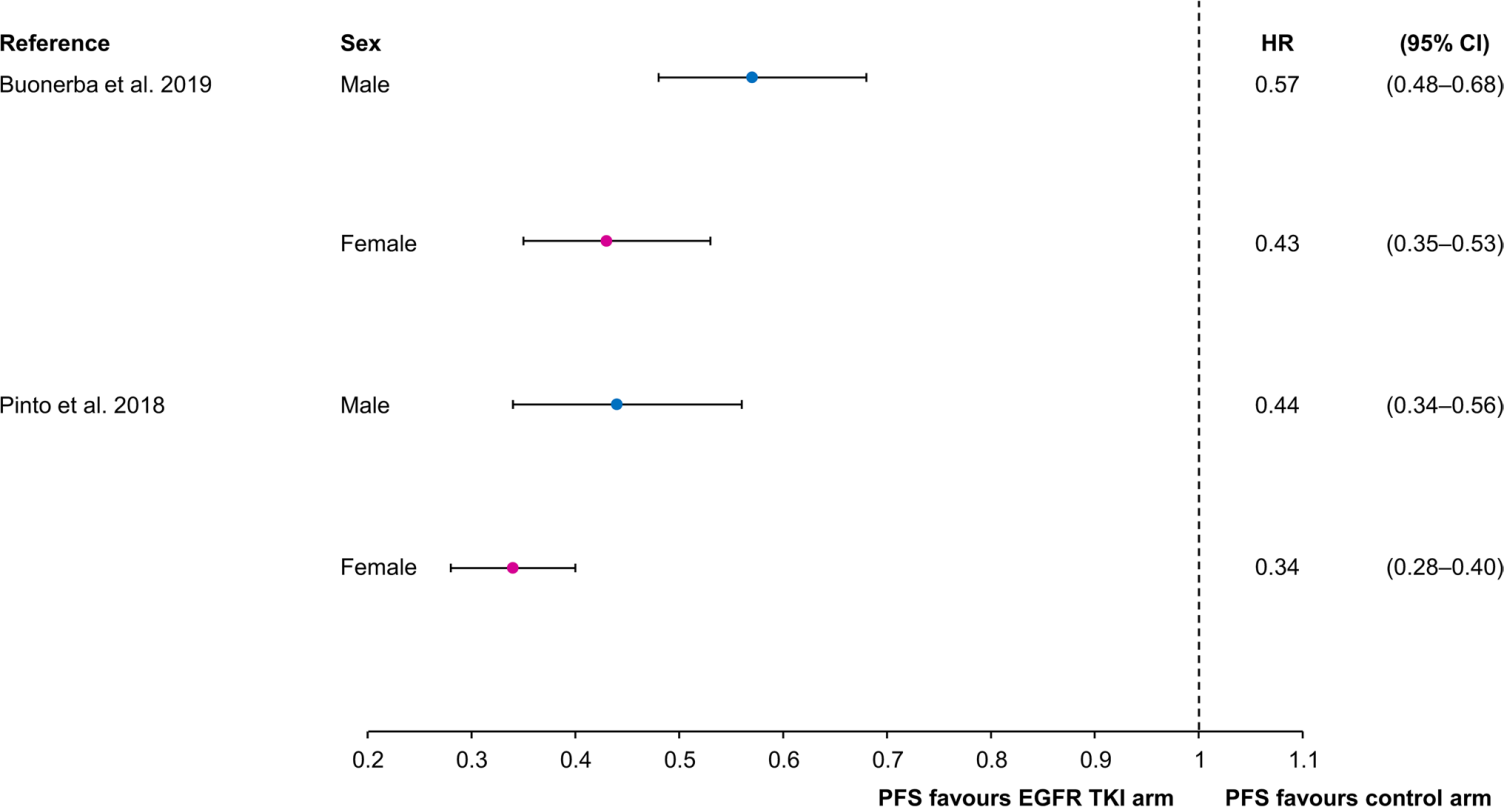
Hazard ratios for progression-free survival in meta-analyses of endothelial growth factor receptor tyrosine kinase inhibitors among patients with non-small cell lung cancer [37, 38]. *CI* confidence interval, *EGFR* endothelial growth factor receptor, *HR* hazard ratio, *PFS* progression-free survival, *TKI* tyrosine kinase inhibitor

There is consistent evidence across a number of meta-analyses that female NSCLC patients usually have better outcomes in response to epidermal growth factor receptor (EGFR) tyrosine kinase inhibitors (TKIs) than male patients, with regard to OS [36] and PFS [37, 38]. Both sexes derived a significant PFS benefit with EGFR TKIs compared with comparators, but the magnitude of the benefit was greater in females than in males (Fig. 2) [37, 38]. The magnitude of the PFS benefit in females was estimated to be about 20% (pooled PFS HR in males vs females of 1.2 [95% CI 1.12–1.56]; *p* = 0.0011) [37]. The PFS benefit in favor of females was consistent whether the EGFR TKI was a first-, second-, or third-generation agent, and whether the control arm was a first-generation EGFR TKI or another form of therapy [37]. Considerable OS benefit was found with EGFR TKIs in females, with a 27% reduction in the risk of death versus males (HR 0.73 [95% CI 0.67–0.79]; *p* < 0.00001), although a high statistical heterogeneity between cohorts was observed [38]. In a subsequent meta-analysis, the largest of these meta-analyses to date (*n* = 11,874), OS was significantly longer in female than male NSCLC patients who received EGFR TKI therapy (HR 0.86 [95% CI 0.75–1.00]; *p* = 0.04) [36].

Notably, no differences in outcomes between males and females have been shown for anaplastic lymphoma kinase (ALK) inhibitors in patients with NSCLC [38], or for TKIs (e.g., imatinib) used to treat patients with chronic myeloid leukemia [39, 40].

### Radiotherapy

There is some evidence that the radiosensitivity of cells (both healthy and cancerous) differs between males and females [41, 42]. The risk of developing certain radiation-associated cancers (particularly thyroid and breast cancer) is higher in premenopausal females than in age-matched males, suggesting that hormonal status is integral to the risk [41]. Additional support for a relationship between hormonal status and risk of radiation-associated cancers is provided by the observation that the organs most susceptible to radiation-induced cancer variy by age in females. In young females (< 50 years of age at exposure), the most common organs affected (in rank order) are the breast, lung, thyroid, and colon, whereas in older females, they are the lung, bladder, colon, and blood (leukemia). The latter is similar to the rank order in males (colon, lung, leukemia, and bladder), which does not change significantly by age [41].

Regarding sex-related differences in effectiveness of radiotherapy, there are relatively few clinical data comparing outcomes in male and female patients [42]. Fewer than half of the studies investigating radiotherapy in cancer patients include sex as a variable in the data analysis [11]. Where data are available, they suggest that female patients achieve greater survival benefits from radiotherapy compared with male patients [42–44]. This may be related to dosage, as women may receive higher amounts of ionizing radiation as a percentage of body weight [45]. For example, a study in patients undergoing definitive radiotherapy for esophageal squamous cell cancer found that sex was a significant independent prognostic factor for OS (HR 0.755 [95% CI 0.615–0.926]; *p* = 0.007) and PFS (HR 0.746 [95% CI 0.611–0.910]; *p* = 0.004) [44]. After propensity score matching to account for potential confounders, female patients had longer OS (median 19.6 vs 16.1 months) and PFS (median 13.5 vs 11.6 months) versus male patients [44]. Female patients also had a significantly lower risk of recurrence (*p* < 0.0009) and distant metastases (*p* = 0.0064). Similarly, an analysis of survival outcomes in patients with head and neck cancer showed better 5-year survival rates in female than male patients who underwent radiotherapy (63.78% vs 57.04%, respectively) and in those who underwent radiotherapy combined with platinum-based chemotherapy (37.23% vs 30.99%) [43]. The differences were not statistically significant, but the authors controlled for confounders using propensity score matching based on age, lymph node status, and nicotine and alcohol dependence [43]. A study in patients with Merkel cell carcinoma reported significantly better OS rates among female than male patients receiving treatment (*p* = 0.04), and among those who received adjuvant radiotherapy versus surgery alone (*p* = 0.0001) [46]. However, in the cohort of patients receiving adjuvant radiotherapy, there was no significant difference in outcomes between males and females [46]. The study may have been underpowered to detect sex-related differences in the radiotherapy subgroup, since Merkel cell carcinoma is a rare disease.

Sex-related differences in body composition and hormonal status can affect the pharmacokinetics and pharmacodynamics of radiopharmaceuticals (such as those used to treat thyroid cancer) and, therefore, radionuclide accumulation; yet, studies into sex-stratified dosimetry are limited [41]. There is also a paucity of data to evaluate whether patient responses to radiosensitizers—agents that enhance the cytotoxic effects of radiotherapy—may differ according to sex [47]. Regarding palliative radiotherapy, sex does not appear to influence survival outcomes in patients with NSCLC [48, 49]. Overall, further research is needed to confirm and understand the influence of sex on the efficacy of radiotherapy, though available evidence suggests that sex-based differences in radiosensitivity may be present. Given that current radiotherapeutic guidelines and treatment protocols are informed by population averages rather than demographic or biological properties, these early findings highlight an opportunity to optimize radiotherapy based on sex in future clinical practice [42].

## Toxicity

**Fig. 3:**
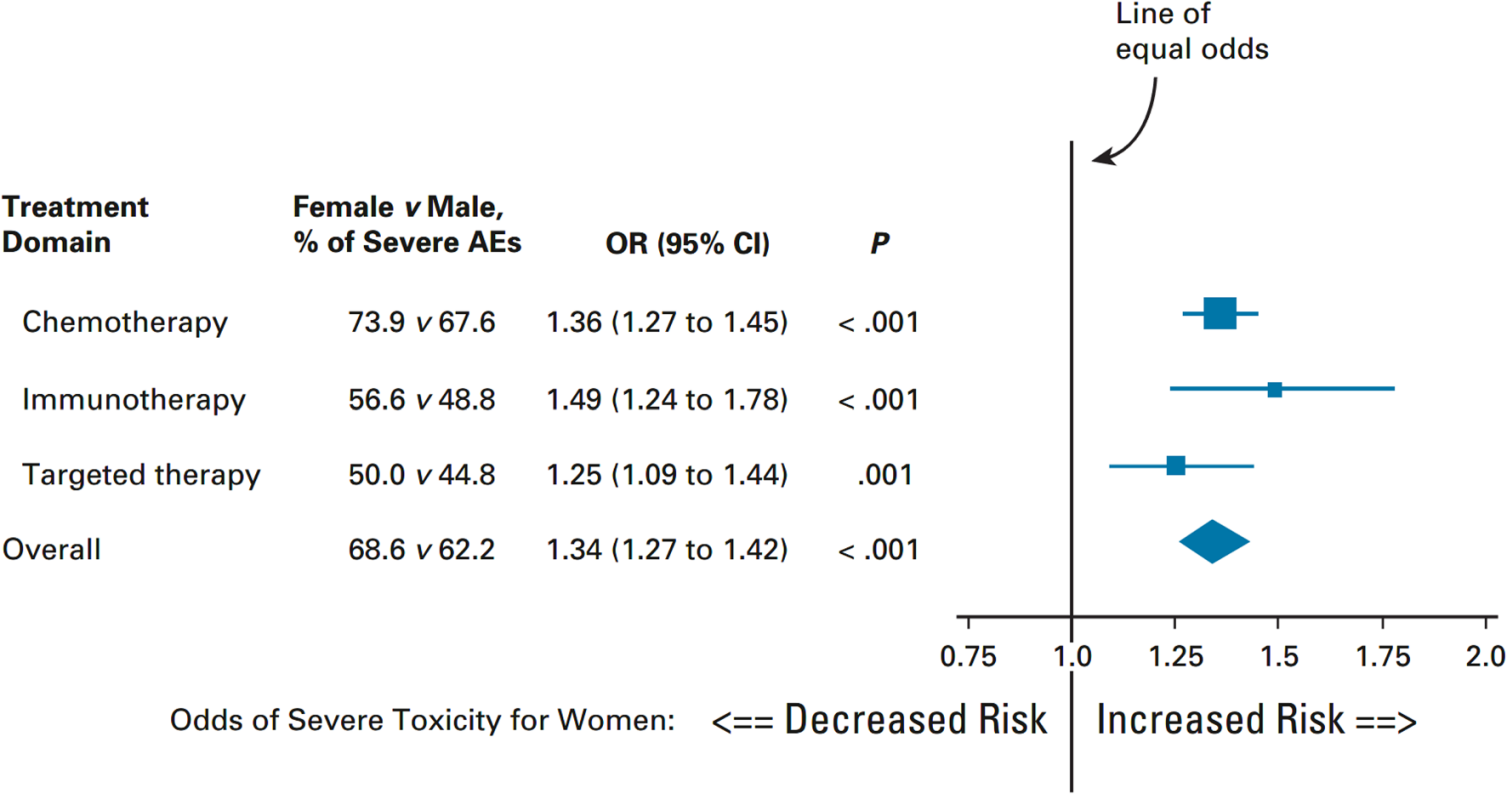
Odds ratios for developing grade ≥ 3 toxicity during treatment for gastroesophageal cancer in females versus males, stratified by type of treatment [30]. *AE* adverse event, *CI* confidence interval, *OR* odds ratio. Reproduced with permission: [50]

Females are at higher risk of grade ≥ 3 toxicity across a range of cancer treatments, including cytotoxic chemotherapy, ICIs, and targeted therapy, with risks increased by 34%, 49%, and 25%, respectively (Fig. 3) [50]. Because recommended doses of drugs are established predominantly in males during clinical trials, females may experience greater systemic drug exposure due to differences in body composition/body surface area and pharmacokinetics [7, 51, 52]. For example, increased drug exposure can result from differences in drug clearance, a larger volume of distribution in women, and/or a larger free fraction in women [53]. In addition, pharmacodynamic factors may play a role, making women more sensitive to drug effects; these factors include receptor number, receptor binding, and signal transduction following receptor binding [53].

Gender may also play a role in the differences in reported AEs. If women are more adherent to treatment, they may experience more AEs as a result of drug exposure; however, AEs are also a common reason for drug discontinuation and therefore poor adherence, so this relationship is complex [54]. Women are more likely than men to report AEs to the physician, as research shows that AE reporting may be influenced by gender norms, such as being ‘stoic’ [55].

### Immunotherapy

**Fig. 4:**
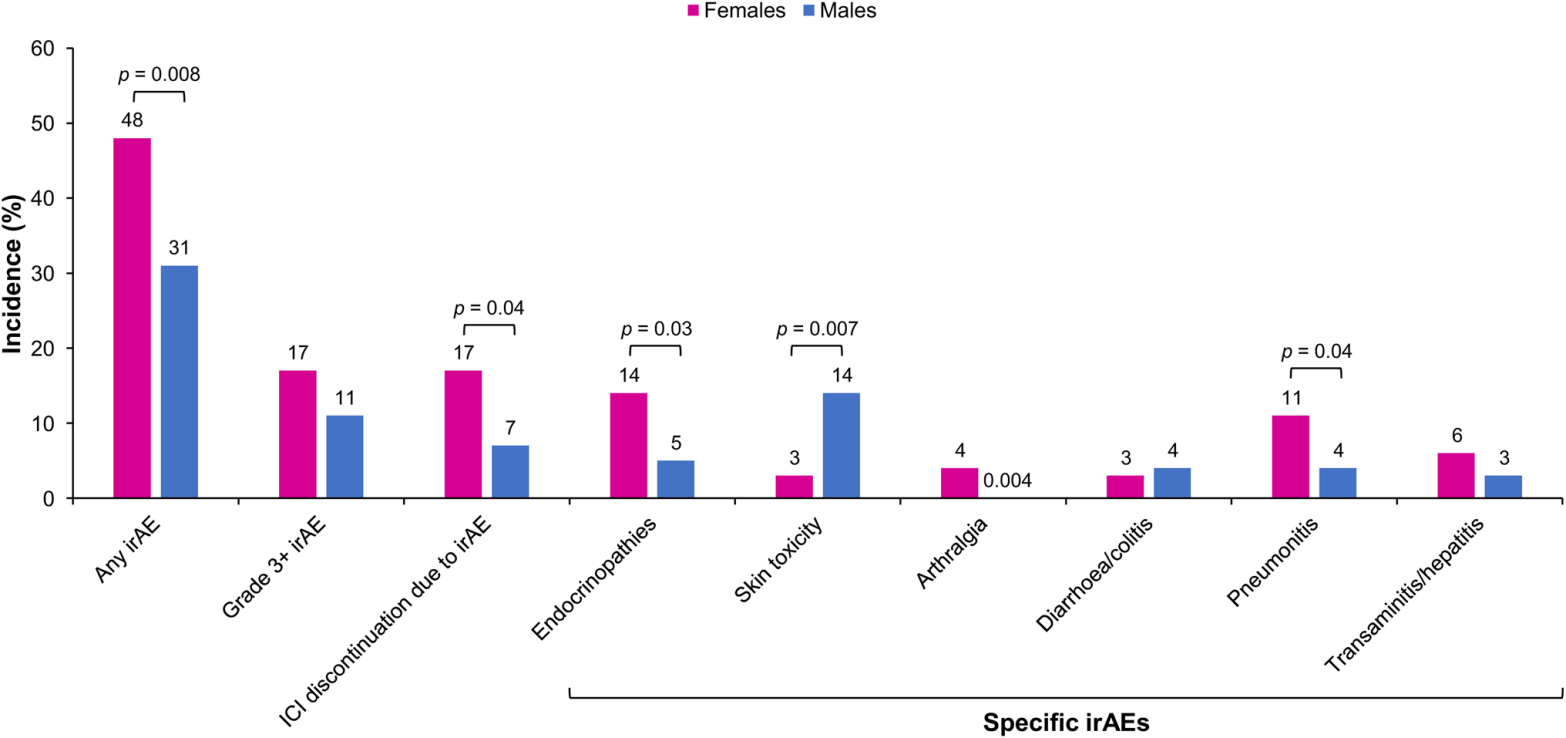
Incidence of immune-related adverse events during treatment with immune checkpoint inhibitors in male and female patients with non-small cell lung cancer [30]. ICI immune checkpoint inhibitor, irAE immune-related adverse event

The relationship between sex and immune-related AEs (irAEs) during immunotherapy is complex, with some researchers showing an increased risk in females [56] and others finding no sex differences [57]. The relationship between sex and irAEs may be influenced by hormonal status. In patients with NSCLC, premenopausal females experienced a higher incidence of irAEs during anti-programmed cell death protein 1 (PD-1) therapy (67%) compared with postmenopausal females (60%) and males (40%) [56]. In addition, there were sex-related differences in the types of irAEs that developed during anti-PD-1 therapy, with females more likely to develop endocrinopathies or arthritis, and males to develop skin toxicity (Fig. 4) [56].

However, these differences may be masked by different treatment patterns between males and females, such as the observation that females are more likely than males to receive steroids during ICI therapy [23]. Moreover, in the case of rare forms of irAEs (such as myocarditis), differences may be influenced by sex bias in treatment decisions or clinical trial recruitment [58]. The risk of cytopenia after CAR-T cell therapy may be lower in males than females [59], although studies are not consistent about a sex interaction in this setting [60].

### Cytotoxic chemotherapy

Across many common types of chemotherapy, the incidence of AEs is higher in females than males. For example, in an analysis of pooled data from four randomized controlled trials in patients with gastroesophageal cancer, females tended to have a higher incidence of most types of AEs (nausea and vomiting, diarrhea, stomatitis, alopecia, grade ≥ 3 neutropenia, and febrile neutropenia) as well as serious AEs, but males had a higher incidence of peripheral neuropathy [30]. However, when adjusted for potential confounders, only grade ≥ 3 gastrointestinal toxicities were significantly associated with female sex (adjusted odds ratio [OR] 1.50 [95% CI 1.07–2.12]; *p* = 0.02) [30].

The relationship between sex and AEs can also differ by age, particularly with regard to anthracycline cardiotoxicity [58, 61]. Studies suggest that among people who receive anthracyclines during childhood for pediatric cancer, the risk of later developing cardiotoxicity is significantly higher in females than males, although this is not a consistent finding across all studies [61]. On the other hand, when patients receive anthracyclines during adulthood, the risk of cardiotoxicity is higher in males than premenopausal females, and similar between males and postmenopausal females [61].

As described above, pharmacokinetic and pharmacodynamic differences may account for some of the sex-related differences in efficacy and toxicity, particularly for some types of treatments (such as the fluoropyrimidines and radionuclides [29, 35, 41, 62]), with higher exposure in females than males [52]. For example, female patients with colorectal cancer experience significantly higher rates of neutropenia, leukopenia, nausea or vomiting, stomatitis, and diarrhea than males during adjuvant therapy with a fluoropyrimidine-containing regimen [63].

### Targeted therapies

There is currently limited information about sex-related differences in the toxicity profiles of targeted therapies; however, available data suggest that toxicity varies by the signaling pathway being targeted [52].

The ADAURA trial reported on osimertinib, a third-generation EGFR TKI, in patients with resected EGFR-mutated NSCLC [64]. There were no between-group differences between males and females in the incidence of AEs (99% [*n* = 108/109] and 97% [*n* = 222/228], respectively) or serious AEs (22% [*n* = 24/109] and 19% [*n* = 44/228]), and the incidence of AEs leading to discontinuation or dose interruption was also similar between sexes. While the incidence of grade ≥ 3 AEs in the osimertinib group was slightly higher in males versus females (27% vs 22%, respectively), AEs leading to dose reduction of osimertinib were more frequent in females than in males (15% vs 6%) [64].

### Radiotherapy

Preclinical data suggest that female hormones may protect against radiation-induced cardiotoxicity [65], but the female sex may exhibit enhanced susceptibility to arthrofibrosis after radiation [66]. Unfortunately, clinical studies examining sex-related differences in radiation toxicity are lacking, but the available data do suggest greater susceptibility in females than males [42]. For example, in a study of children with acute lymphoblastic leukemia who underwent cranial radiotherapy before the age of 4 years, the intelligence quotient (IQ) deficit later in childhood (age ~ 7 years) was greater in female than male patients [67]. The effect of sex was most notable for impairment of verbal IQ, with lower scores in female patients who had received treatment aged < 4 years (mean ± standard deviation [SD] scores of 86.8 ± 11.1 vs 96.5 ± 12.5 in age-matched male patients) [67]. Females may be more susceptible to the toxicity of radiochemotherapy; a pooled analysis of three studies in patients with advanced rectal cancer found that females had significantly more profound lymphopenia (reduction in epithelial CD8 + cytotoxic lymphocytes) and experienced worse quality of life related to nausea, vomiting, diarrhea, and loss of appetite during radiochemotherapy compared with males [45]. In addition, a meta-analysis of ten studies in patients undergoing radiotherapy for Hodgkin’s lymphoma identified a greater risk of radiation-related cardiovascular events and mortality among females than males (OR 3.74 [95% CI 2.44–5.72]; *p* < 0.0001), as well as an increased risk of all-cause mortality (OR 1.94 [95% CI 1.10–3.44]; *p* < 0.023) [68]. The authors observed that most of the studies included in their analysis contained a paucity of data relating to total radiation dose received, frequency and duration of radiation administered, side of chest irradiated, chemotherapy administered during radiation treatment, and presence of cardiovascular risk factors [68]. This highlights the need not only for cardiovascular disease screening among at-risk patients, especially female patients, but also further research to understand possible differences in treatment profiles that may help explain observed differences in toxicity.

## Conclusion

Current data suggest that sex affects treatment efficacy and toxicity outcomes among patients with cancer, and that sex should be included as an important variable when individualizing treatment. However, sex-related differences may be influenced by a number of potential confounders, including the average age at diagnosis of specific cancers [8] and the under-representation of females in clinical trials [12]. In addition, many studies do not include sex as a variable in data analyses [11]. There remains a paucity of studies examining the impact of gender on the outcomes of cancer treatment.

We recommend greater research into sex- and gender-related differences in treatment efficacy and toxicity, and the factors that may modify these differences, to truly personalize cancer treatment. We welcome initiatives by cancer organizations, such as the Spanish Society of Medical Oncology (SEOM) and European Society for Medical Oncology (ESMO), to promote such research and address some of the inherent methodological challenges [5, 69].

## Data Availability

Data sharing is not applicable to this article as no datasets were generated or analyzed during the course of this work.
